# The independent contribution of executive functions to health related quality of life in older women

**DOI:** 10.1186/1471-2318-10-16

**Published:** 2010-04-01

**Authors:** Jennifer C Davis, Carlo A Marra, Mehdi Najafzadeh, Teresa Liu-Ambrose

**Affiliations:** 1 Centre for Hip Health & Mobility, University of British Columbia & Vancouver Coastal Health Research Institute (VCHRI), 301-2647 Willow Street, Vancouver, British Columbia, V5Z 3P1, Canada; 2 Faculty of Pharmaceutical Sciences, University of British Columbia, Vancouver, British Columbia, Canada; 3 Collaboration for Outcomes Research and Evaluation, St Paul's Hospital, 620B 1081 Burrard Street, University of British Columbia, Vancouver, British Columbia, V6Z 1Y6, Canada

## Abstract

**Background:**

Cognition is a multidimensional construct and to our knowledge, no previous studies have examined the independent contribution of specific domains of cognition to health related quality of life. To determine whether executive functions are independently associated with health related quality of life assessed using Quality Adjusted Life Years (QALYs) calculated from the EuroQol EQ-5D (EQ-5D) in older women after adjusting for known covariates, including global cognition. Therefore, we conducted a secondary analysis of community-dwelling older women aged 65-75 years who participated in a 12-month randomized controlled trial of resistance training. We assessed global cognition using the Mini-Mental State Examination (MMSE) and executive functions using the: 1) Stroop Test; 2) Trail Making Test (Part B) and 3) Digits Verbal Span Backwards Test. We calculated QALYs from the EQ-5D administered at baseline, 6 months and 12 months.

**Results:**

Our multivariate linear regression model demonstrated the specific executive processes of set shifting and working memory, as measured by Trail Making Test (Part B) and Digits Verbal Span Backward Test (p < 0.01) respectively, were independently associated with QALYs after accounting for age, comorbidities, general mobility, and global cognition. The final model explained 50% of the variation in QALYs.

**Conclusions:**

Our study highlights the specific executive processes of set shifting and working memory were independently associated with QALYs -- a measure of health related quality of life. Given that executive functions explain variability in QALYs, clinicians may need to consider assessing executive functions when measuring health related quality of life. Further, the EQ-5D may be used to track changes in health status over time and serve as a screening tool for clinicians.

**Trial Registration:**

ClinicalTrials.gov Identifier: NCT00426881.

## Background

Health related quality of life (HRQL) is an important construct that describes an individual's overall health status. It is commonly used in economic evaluations [1] as a measure of health benefit, and may be more responsive among populations with conditions associated with high morbidity [2]. HRQL is defined by several domains [3], with general agreement that emotion, physical and social are core domains. These concur with WHO's definition of health - a state of complete physical, mental, and social well-being, and not merely the absence of disease or infirmity [4]. However, the specific contribution of HRQL to quality of life remains unknown [5] given HRQL is "the subjective assessment of the impact of disease and treatment across the physical, psychological, social, and somatic domains of functioning and well-being [6]."

The use of a generic, preference-based instrument is one method commonly used to assess HRQL [7]. The EQ-5D is one example of such a generic preference based utility instrument developed by the EuroQol Group [8]. The EQ-5D captures 243 unique health states and captures the following domains using a short five-item questionnaire: 1) mobility, 2) self-care, 3) usual activities, 4) pain and 5) anxiety/depression [8]. Individuals' preferences for the scoring of the EQ-5D were estimated using the time trade off technique on a random sample of adults taken from the population living in the York (UK) region (N = 3000) [9]. The EQ-5D is the most widely used generic instrument that uses a utility-based scoring approach, yielding a single summary score on a common scale to facilitate comparison across different health conditions and patient populations [10,11]. The single summary score, defined as a health state utility value (HSUV) is anchored at zero - a health state equivalent to death and 1.0 - a state of "full health." HSUVs less than zero are defines health states worse than death.

HSUVs are used to calculate Quality Adjusted Life Years (QALYs) to account for the quality of life of a patient (measured using health utilities from a generic preference based utility instrument such as the EQ-5D) in a given health state and the time spent in that health state. Briefly, the QALY is a useful measure of health benefit because it simultaneously captures both quantity and quality gains or losses [12]. A key benefit of the QALY is that it enables direct comparison of patient outcomes across diseases and diverse health interventions [12]. Also, it accounts for changes in both morbidity and mortality under a common metric. QALYs are defined as a measure of health benefit in terms of time spent in a series of quality-weighted health states, in which the quality weights reflect the desirability of living in the state, typically anchored at "perfect" health (weighted 1.0) to dead (weighted 0.0)" [13]. The quality weights spent in each state are multiplied by the time spent in each state. The sum of all these products is the total number of QALYs. QALYs are one measure used to assess HRQL.

Clinical measures that are associated with HRQL include cognition, physical disability and chronic conditions such as rheumatoid arthritis, sex, social functioning and physical activity [14]. Specifically, two studies demonstrated that adults with a physical disability, asthma or who are female had significantly increased odd ratios for poor HRQL [15,16]. Among individuals with Alzheimer's disease, global cognition as measured by the Mini Mental State Examination (MMSE), was associated with HSUVs measured by the EQ-5D [17].

Cognition is a multidimensional construct and to our knowledge, no previous studies have examined the independent contribution of specific domains of cognition to HRQL. We hypothesize that executive functions may be of particular importance to HRQL. Executive functions are higher-order cognitive processes that control planning, initiation, sequencing and monitoring of complex goal directed behavior [18,19]. These cognitive processes are essential to the person's ability to carry out health-promoting behaviours [20], such as medication management, dietary and lifestyle changes, self-monitoring of responses, and follow-up with health care professionals. Hence, in this study, we examined whether executive functions are independently associated with HRQL in community-dwelling older women, calculated using the EQ-5D HSUVs at three time points, after accounting for global cognitive function and known covariates.

## Methods

### Study Design and Participants

The total sample for this analysis consisted of 135 women who consented and completed a randomized controlled trial of exercise (NCT00426881; Brain Power study) that aimed to examine the effect of once-weekly and twice-weekly resistance training on cognitive performance of executive functions. The design and the primary results of the Brain Power study have been reported elsewhere [21]. Briefly, participants enrolled in Brain Power were: aged 65 to 75 years, community-dwelling, and had a MMSE ≥ 24.

### Functional Comorbidity Index

Functional Comorbidity Index was calculated to estimate the degree of comorbidity associated with physical functioning [22]. This scale's score is the total number of comorbidities.

### Global Cognition Measures - Mini Mental State Examination

The MMSE is a widely used and well-known questionnaire used to screen for cognitive impairment (i.e., MMSE <24) [23]. It is scored on a 30-point scale with a median score of 28 for more normal octogenarians with more than 12 years of education [23]. The MMSE may underestimate cognitive impairment for frontal system disorders [24] because it has no items specifically addressing cognitive function [23].

### Central Executive Functions--Set Shifting, Updating and Response Inhibition

Our assessment of executive functions were composed of three tests that measure different aspects of executive functions: [18] 1) Trail Making Test Part B, 2) Verbal Digits Backward Test and 3) Stroop Colour-Word Test.

#### Trail Making Part B

We used the Trail Making Part B test to assess set shifting. Set shifting refers to an individuals ability to go back and forth between multiple tasks or mental sets [25]. The test consists of one page with circled letters (A-L) and numbers (1-13). We instructed participants to draw a single line as quickly and accurately as possible from 1 to A, A to 2, 2 to B and so forth until the task was completed. We recorded the number of errors and the length of time the task took. To index set shifting, we calculated the difference between Part B and Part A completion time. Smaller difference scores indicate more cognitive flexibility. Reliability scores for the Trail Making Part B varied from moderate to excellent [26].

#### Verbal Digits Backward

We used the Verbal Digits Backward test to assess working memory [27]. Working memory (updating) refers to an individuals ability filter incoming information for relevance to the current task and subsequently update informational content replacing old non-relevant information with new relevant incoming information [25]. Seven pairs of random number sequences were read aloud by an assessor at one number per second. The first sequence of numbers is three and the sequence was increased by one number up to a length of nine digits. Participants repeated each sequence in exactly the reverse order until they failed two attempts of the same sequence length. It was scored on a 14-point scale with higher scores indicating a better performance. For the verbal digits forward test, the participant's task is to repeat each sequence exactly as it is given. The difference between the verbal digits forward test score and the verbal digits backward test score was used as an index of the central executive component of working memory. Smaller difference scores indicate better working memory.

#### Stroop Test

The Stroop Test, assessed response inhibition [28] including deliberate inhibition of automatic, dominant or routine responses [26]. For the primary test condition, participants were presented colour-words printed in incongruent coloured inks (e.g., the word "BLUE" printed in red ink) and were required name the ink colour that the words were printed while ignoring the word itself. We recorded the time participants took to read each condition. The ability to selectively attend and control response output was calculated as the time difference between the test condition and the priming condition (e.g., coloured X's). Smaller time differences indicate better selective attention and conflict resolution.

### Preference Based Measures - HSUV Instrument

The HSUV instrument we used was the EQ-5D. Major differences between the EQ-5D and other preference based measures were outlined previously [8]. The EQ-5D does not directly measure cognition; another generic preference based instrument, the Health Utilities Index Mark 3 (HUI3) does [29]. To our knowledge, no previous studies have examined the association between executive functions and HRQL using the HUI3. Therefore, we chose the EQ-5D given that is it the most widely used generic preference based utility instrument that has been used among individuals with cognitive decline.

The EQ-5D is a short five item generic HSUV instrument designed to assess HRQL [30]. The EQ-5D short structure was considered a strength in terms of high response rates, participant burden and feasibility [31] and a weakness in terms of its responsiveness and sensitivity [32]. The EQ-5D is used for cross-national comparisons of health status [33] and captures 243 unique health states [8]. We used the EQ-5D to calculate QALYs as an assessment of an individual's HRQL according to the following five EQ-5D domains: mobility, self-care, usual activities, pain, and anxiety/depression. Each domain has three possible options that either indicates no problems, some problems or severe problems. The EQ-5D HSUVs at each time point are bounded from -0.54 to 1.00 where a score of less than zero is indicative of a health state worse than death. We used three HSUVs for each individual from the EQ-5D at baseline, 6 months and 12 months to calculate QALYs for each individual. Specific to this study only, QALYs are a measure of HRQL because zero participants died and all participants were followed for the same time period, thus any changes in QALYs are due to quality of life, rather than quantity of time spent in a given health state.

### Timed Up and Go

We used the Timed Up and Go Test (TUG) to assess general mobility [34]. Participants were instructed to rise from a chair with their arms crossed (seat height 45 cm), walk a distance of three meters, turn around, walk back to the chair, and sit down with their arms crossed around their chest. We timed each trial and took the mean of two trials for our statistical analysis.

### Data Analysis

We analysed all data using STATA version 10.0. Our base case analysis included 135 women based on recommendations for multiple imputation of missing cost and HSUV data [35]. For all discrete time points, we used a combination of multiple imputation and bootstrapping to estimate uncertainty caused by missing values and we report both the imputed data set analysis and a complete case analysis. Our complete case analysis consisted of 89 participants for the EQ-5D who had all three HSUVs at baseline, 6-months and 12-months.

We report descriptive data for all variables of interest. For data that are normally distributed we report mean and standard deviation and frequencies depending on the measure. For data that were significantly skewed, we report median and interquartile range. We used the Pearson product moment correlation coefficient to determine the level of association between QALYs and age, group, education, average waist girth, functional comorbidity index, general mobility, global cognition and executive functions.

In our multiple linear regression model, age, group, education, average waist girth, functional comorbidity index, general mobility and global cognition were statistically controlled by forcing these six variables into the regression model first (Model 1). These independent variables were determined based on the results of the Pearson product moment coefficient analyses (i.e., alpha level ≤ 0.05) and assumed biological relevance, such as MMSE and waist girth were entered into the model regardless of the results of the correlation analyses. Each of the executive functions (i.e., Trail Making Part B, Digits Backwards, Stroop Colour Word) was then entered sequentially into the model. Those that significantly added to the model (i.e., significant change in R^2^) were kept in the model. Digits Backward was entered last into the model. We assessed the assumptions of normality of the residuals and heteroscedasticity.

## Results

We report the results of both the imputed case analysis and the complete case analysis. For the complete case analysis, we calculated QALYs from the EQ-5D for 89 of the 135 participants.

### Sample

Table 1 reports descriptive statistics for descriptive variables (age, baseline EQ-5D HSUV, group, education, average waist girth, functional comorbidity index, trail making part A, trail making part B, Digits Forward, MMSE and TUG) and our outcome of interest (QALYs). Participants included in our imputed and case analysis were similar on demographic characteristics. Overall, this cohort of community-dwelling senior women were high functioning individuals as indicated by their baseline EQ-5D HSUVs of 0.82 (SD: 0.19) and 0.85 (SD: 0.18) for the imputed and complete case sets, respectively. Further, the mean MMSE was greater than 28 (max 30 points).

**Table 1:** Characteristics of the Brain Power cohort at baseline (N = 89)

Variable at Baseline	Imputed Data Set		Complete Case Set	
	**Mean**	**Standard Deviation**	**Mean**	**Standard Deviation**

QALY (EQ-5D)	0.83	0.17	0.83	0.17
Age (years)	69.6	3.0	69.7	3.0
Baseline EQ-5D HSUV	0.82	0.19	0.85	0.18
Average waist girth (cm)	86.3	13.0	87.3	12.4
Function Comorbidity Index	2.1	1.7	2.0	1.6
Trail A (sec)	55.3	18.3	54.4	17.7
Trail B (sec)	101.2	41.7	97.0	36.7
Trail B - Trail A	42.5	29.9	46.2	34.8
Digits Forward (max 14 pts)	7.9	2.3	7.9	2.3
Digits Backward (max 14 pts)	4.5	2.4	4.3	2.4
Digits Forward - Digits Backward	3.7	2.3	3.4	2.3
MMSE (max 30 pts)	28.6	1.3	28.7	1.4
Timed Up and Go Test (sec)	6.6	1.4	6.7	1.5

### Correlation Coefficients

Table 2 reports the correlation coefficients between variables of interest and QALYs. Age, education, baseline EQ-5D HSUV, average waist girth, functional comorbidity index, TUG, set shifting (assessed by the difference score for Trail Making Part B and A) and working memory (assessed by the difference score for Digits Forward and Backward) were significantly associated with QALYs calculated from the EQ-5D (p < 0.05). Group and response inhibition (assessed by using the Stroop Colour-Word test) were not significantly associated with QALYs calculated from the EQ-5D (p > 0.05).

**Table 2:** Correlation coefficient matrix^‡^

Variable at Baseline	Imputed Data Set QALYs (EQ-5D)
Age	-0.298**
Group	0.0913
Education	0.3106**
Average waist girth	-0.232*
Function Comorbidity Index	-0.488**
Trail B - Trail A	-0.1084*
Digits Forward - Digits Backward	-0.1710**
MMSE	0.051
Timed Up and Go	-0.598**
Stroop	-0.113

^‡ ^Results from both imputed and complete case analysis were identical to 0.000 decimal.

* p < 0.05

** p < 0.01

### Multivariate Linear Regression Results for QALYs calculated from the EQ-5D

The Trail Making Part B was a significant and independent predictor for HRQL as assessed by the EQ-5D (p < 0.01). Digits Backward was also a significant predictor for HRQL based on the EQ-5D (p < 0.01) based on the results of the imputed data set (for complete case set, p = 0.09). Adding the Trail Making Part B and the Digits Backward resulted in an R^2 ^change of 4% (p < 0.01). The total variance accounted for by our final model was 50% (Table 3). The R^2 ^and R^2 ^change from both imputed and complete case analysis were identical to 1/100 decimal. The Stroop Colour-Word task did not significantly improved the model after accounting for age, group, education waist girth, functional comorbidity index, general mobility and global cognition.

**Table 3:** Bivariate and Multiple Linear Regression Summary for QALYs in Older Women Calculated from EQ-5D HSUVs^‡^

	Imputed Data Set		Complete Case Set	
**Independent Variables**	**Unstandardized ß (Standard Error)**	**P-value**	**Unstandardized ß (Standard Error)**	**P-value**

Model	R^2 ^0.536		R^2 ^0.536	
Trail B - Trail A	0.0012 (0.0002)	0.00**	0.0012 (0.0005)	0.031*
Digits Forward -Digit Backward	-0.011 (0.003)	0.00**	-0.011 (0.007)	0.084
Age	0.0008 (0.0022)	0.717	0.0008 (0.0052)	0.88
Group	-0.008 (0.008)	0.296	-0.008 (0.018)	0.66
Education	0.025 (0.005)	0.00**	0.02 (0.01)	0.024*
Average waist girth	-0.0006 (0.0005)	0.240	-0.0006 (0.0012)	0.62
Functional Comorbidity Index	-0.036 (0.004)	0.00**	-0.036 (0.009)	0.00**
Timed Up and Go	-0.062 (0.005)	0.00**	-0.06 (0.01)	0.00**
MMSE	-0.017 (0.005)	0.001	-0.02 (0.01)	0.15

^‡ ^R^2 ^and R^2 ^change were the same for both imputed and complete case analysis

* p < 0.05

** p < 0.01

## Discussion

### Relationship between executive functions and QALYs - HRQL

Persons who experience cognitive decline have a reduced quality of life [36]. To our knowledge, our study is the first to demonstrate the independent association between key executive processes as measured by standard neuropsychological tests, and QALYs measured prospectively over one year among high functioning community-dwelling senior women. Of particular importance, this independent association was found in this cohort of senior women after accounting for age, waist girth, functional comorbidity index, general mobility and global cognition. Also, our final model explained 50% of the variation in QALYs; regression models in clinical research often do not account for such a large amount of variance [37].

We specifically found that, both set shifting and working memory, were independently associated HRQL, measured by QALYs calculated from the EQ-5D HSUVs. Our novel result extends previous findings that set shifting, as the Trail Making B Test, is associated with factors that may influence QALYs: 1) mobility [38,39]; 2); medication adherence [40]; 3) driving performance [41]; 4) anxiety and emotional regulation [42]. We highlight that mobility and anxiety/depression are domains in the EQ-5D. Additionally, our current finding also extends previous findings that working memory is associated with pain severity; pain is one of the five domains of the EQ-5D; therefore, we would expect an association with QALYs [43].

We acknowledge that executive functions are only one aspect of cognition and were the sole cognitive processes explored in the Brain POWER study [21]. The Brain POWER study focused specifically on executive functions because these cognitive processes: 1) decline substantially with aging [44]; 2) are associated with the ability to carry out health-promoting behaviours [45]; and 3) are most responsive to exercise training [46]. Because executive functions are associated with the ability to carry out health-promoting behaviours [45], we hypothesized that reduced executive functioning may directly impact the overall health status of older adults. However, we acknowledge that other cognitive domains may also influence health status. Hence, future studies are needed to explore the contribution of other cognitive domains to health status in older adults.

### Establishing a relationship between working memory and health related quality of life

Our finding of both set shifting and working memory contributing to health related quality of life concur and extend previous studies examining the association of cognitive function and instrumental activities of daily living. Instrumental activities of daily living include the ability to prepare a balanced meal, remember appointments, keep financial records and take medications as prescribed [47]. Health related quality of life is related to one's ability to perform instrumental activities of daily living [48] and one's overall mobility [49] Previous studies have demonstrated that executive functions are associated with instrumental activities of daily living and functional status among older adults [50,51]. Specifically, the Trail Making B Test is an independent predictor of the instrumental activities of daily living [50,51].

### Response inhibition and health related quality of life - comparison with another study

Our findings for the Stroop test and health related quality of life differ from those of previous research [52]. Specifically, one study conducted among 72 older adults with stable cardiovascular disease found a significant association between response inhibition and instrumental activities of daily living [52]. Differences in the population studied may be a potential reason for our conflicting finding. Participants of the Brain POWER [21] cohort were high functioning individuals. Hence, a ceiling effect for both instrumental activities of daily living and health related quality of life as assessed by the EQ-5D may have existed. Further research is needed to better understand the contribution of response inhibition to health related quality of life.

### Contrasting the imputed and complete case analyses

The lack of a significant association between global cognition and HRQL for our complete case analysis was contrary to the results of our imputed data set analysis. This difference likely was due to the smaller sample size of the complete case analysis. A previous study found a linear relationship between HRQL, assessed using the Assessment of Quality of Life instrument, and MMSE in individuals with Alzheimer's disease -- a finding similar to that of our imputed data set analysis [53]. Further, the "absence of evidence is not evidence of absence [54]." Bland and Altman highlighted study findings that are statistically nonsignificant is not an indication that these findings are indeed nonsignificant or not of clinical important. Rather, because studies lack the necessary power to detect real, and clinically worthwhile, differences in treatment, that we should not interpret or conclude that this is necessarily evidence of no effect. Therefore, because our findings are consistent with one previous study [53], we interpret the discrepant results as a lack of statically power to detect a difference given the smaller sample size in our complete case analysis.

### Timed Up and Go was a key explanatory variable in our model

We found that the TUG [34] was most strongly associated with HRQL in our bivariate analyses, accounting for 27% of the variation in QALYs. One previous study found that functional ability/pain explained most of the variation in global utility score; however, this assessment was not based on a specific measure of mobility such at the TUG [55]. Three previous studies investigated the association between the TUG and the Physical Function domain of the SF-36 [56-58] and two indicated the TUG explained approximately 20% of the variation [57,58]. Therefore, our findings extend previous work with a difference preference based generic utility instrument demonstrating that TUG is strongly associated with EQ-5D HSUVs.

## Conclusions

We note that our small study sample consisted only of older community dwelling women who were cognitively intact; therefore, we cannot say with certainty that these findings are generalizable to older women with mild cognitive impairment or dementia, older men, other age groups and adults who are not community-dwelling. Thus, our study highlights the need for future prospective studies to ascertain whether our present finding apply to other clinical populations and whether changes in executive functions, specifically the cognitive processes of set shifting and working memory are causally linked to changes in HRQL assessed using generic preference based HSUV instruments, such as the EQ-5D. Our findings indicate that EQ-5D HSUVs over time can be largely explained by baseline measures of age, waist girth, functional comorbidity index, general mobility, global cognition and the cognitive processes of set shifting and working memory. Given that set shifting and working memory explain a statistically significant amount of variability in QALYs, clinicians may need to consider assessing these cognitive processes in response to patients perceived health status (i.e., health related quality of life).

## Competing interests

The authors declare that they have no competing interests.

## Authors' contributions

JCD was principal investigator for the evaluation of HRQL and healthcare resource use and, was responsible for design, data analysis, data interpretation, writing of manuscript. TLA was principal investigator for the Brain Power study and was responsible for study concept and design, acquisition of data, data analysis and data interpretation, and reviewing of the manuscript. CAM and MN were responsible for design, data interpretation and critical review of manuscript.

## Pre-publication history

The pre-publication history for this paper can be accessed here:

http://www.biomedcentral.com/1471-2318/10/16/prepub

## References

[B1] Sadatsafavi M Marra CA Ayas NT Stradling J Fleetham J Cost-effectiveness of oral appliances in the treatment of obstructive sleep apnoea-hypopnoea Sleep Breath 2009 13 3 241 252 10.1007/s11325-009-0248-4 19229577

[B2] Sach TH Foss AJ Gregson RM Zaman A Osborn F Masud T Harwood RH Falls and health status in elderly women following first eye cataract surgery: an economic evaluation conducted alongside a randomised controlled trial Br J Ophthalmol 2007 91 12 1675 1679 10.1136/bjo.2007.118687 17585002 PMC2095519

[B3] Matza LS Swensen AR Flood EM Secnik K Leidy NK Assessment of health-related quality of life in children: a review of conceptual, methodological, and regulatory issues Value Health 2004 7 1 79 92 10.1111/j.1524-4733.2004.71273.x 14720133

[B4] World Health Organization Constitution of the World Health Organization Geneva 1948

[B5] Coons SJ Health-related quality of life: let's measure and report it appropriately Clin Ther 2007 29 12 2746 2747 10.1016/j.clinthera.2007.12.031 18201592

[B6] Revicki DA Osoba D Fairclough D Barofsky I Berzon R Leidy NK Rothman M Recommendations on health-related quality of life research to support labeling and promotional claims in the United States Qual Life Res 2000 9 8 887 900 10.1023/A:1008996223999 11284208

[B7] Guyatt GH Feeny DH Patrick DL Measuring health-related quality of life Ann Intern Med 1993 118 8 622 629 8452328 10.7326/0003-4819-118-8-199304150-00009

[B8] Marra CA Woolcott JC Kopec JA Shojania K Offer R Brazier JE Esdaile JM Anis AH A comparison of generic, indirect utility measures (the HUI2, HUI3, SF-6D, and the EQ-5D) and disease-specific instruments (the RAQoL and the HAQ) in rheumatoid arthritis Soc Sci Med 2005 60 7 1571 1582 10.1016/j.socscimed.2004.08.034 15652688

[B9] Dolan P Modeling valuations for EuroQol health states Med Care 1997 35 11 1095 1108 10.1097/00005650-199711000-00002 9366889

[B10] Torrance GW Measurement of health state utilities for economic appraisal J Health Econ 1986 5 1 1 30 10.1016/0167-6296(86)90020-2 10311607

[B11] Torrance GW Utility approach to measuring health-related quality of life J Chronic Dis 1987 40 6 593 603 10.1016/0021-9681(87)90019-1 3298297

[B12] Drummond MF Sculpher MJ Torrance GW O'Brien B Stoddart GL Methods for the economic evaluation for health care programmes 2005 Third New York. United States of America: Oxford University Press

[B13] Neumann PJ Goldie SJ Weinstein MC Preference-based measures in economic evaluation in health care Annu Rev Public Health 2000 21 587 611 10.1146/annurev.publhealth.21.1.587 10884966

[B14] Morey MC Snyder DC Sloane R Cohen HJ Peterson B Hartman TJ Miller P Mitchell DC Demark-Wahnefried W Effects of home-based diet and exercise on functional outcomes among older, overweight long-term cancer survivors: RENEW: a randomized controlled trial JAMA 2009 301 18 1883 1891 10.1001/jama.2009.643 19436015 PMC2752421

[B15] Jiang Y Hesser JE Patterns of health-related quality of life and patterns associated with health risks among Rhode Island adults Health Qual Life Outcomes 2008 6 49 10.1186/1477-7525-6-49 18620582 PMC2481258

[B16] Rabins PV Black BS Measuring quality of life in dementia: purposes, goals, challenges and progress Int Psychogeriatr 2007 19 3 401 407 10.1017/S1041610207004863 17346364

[B17] Jonsson L Andreasen N Kilander L Soininen H Waldemar G Nygaard H Winblad B Jonhagen ME Hallikainen M Wimo A Patient- and proxy-reported utility in Alzheimer disease using the EuroQoL Alzheimer Dis Assoc Disord 2006 20 1 49 55 10.1097/01.wad.0000201851.52707.c9 16493236

[B18] Royall DR Lauterbach EC Cummings JL Reeve A Rummans TA Kaufer DI LaFrance WC Jr Coffey CE Executive control function: a review of its promise and challenges for clinical research. A report from the Committee on Research of the American Neuropsychiatric Association J Neuropsychiatry Clin Neurosci 2002 14 4 377 405 12426407 10.1176/jnp.14.4.377

[B19] Stuss DT Alexander MP Executive functions and the frontal lobes: a conceptual view Psychol Res 2000 63 3-4 289 298 10.1007/s004269900007 11004882

[B20] Kuo HK Lipsitz LA Cerebral white matter changes and geriatric syndromes: is there a link? J Gerontol A Biol Sci Med Sci 2004 59 8 818 826 15345732 10.1093/gerona/59.8.m818

[B21] Liu-Ambrose T Nagamatsu LS Graf P Beattie BL Ashe MC Handy TC Resistance training and executive functions: a 12-month randomized controlled trial Arch Intern Med 2010 170 2 170 178 10.1001/archinternmed.2009.494 20101012 PMC3448565

[B22] Groll DL To T Bombardier C Wright JG The development of a comorbidity index with physical function as the outcome J Clin Epidemiol 2005 58 6 595 602 10.1016/j.jclinepi.2004.10.018 15878473

[B23] Folstein MF Folstein SE McHugh PR "Mini-mental state". A practical method for grading the cognitive state of patients for the clinician J Psychiatr Res 1975 12 3 189 198 10.1016/0022-3956(75)90026-6 1202204

[B24] Royall DR Polk M Dementias that present with and without posterior cortical features: an important clinical distinction J Am Geriatr Soc 1998 46 1 98 105 9434673 10.1111/j.1532-5415.1998.tb01022.x

[B25] Miyake A Emerson MJ Friedman NP Assessment of executive functions in clinical settings: problems and recommendations Semin Speech Lang 2000 21 2 169 183 10.1055/s-2000-7563 10879548

[B26] Spreen O Strauss E A compendium of Neurological Tests, 2nd edition 1998 New York: Oxford University Press

[B27] Wechsler D Wechsler Adult Intelligence Scale--Revised 1981 San Antonio: Psychological Corporation

[B28] Trenerry M Crosson B De Boe J Stroop Neuropsychological Screening Test 1989 Odessa, Florida: Psychological Assessment Resources, Incorporated

[B29] Feeny D Furlong W Torrance GW Goldsmith CH Zhu Z DePauw S Denton M Boyle M Multiattribute and single-attribute utility functions for the health utilities index mark 3 system Med Care 2002 40 2 113 128 10.1097/00005650-200202000-00006 11802084

[B30] Brooks R EuroQol: the current state of play Health Policy 1996 37 1 53 72 10.1016/0168-8510(96)00822-6 10158943

[B31] Brooks R Robin R de Charro F The measurement and valuation of health status using EQ-5D: a European perspective (evidence from the EuroQoL BIOMED research programme) 2003 Netherlands: Kluwer Academic Publishers

[B32] Conner-Spady B Suarez-Almazor ME Variation in the estimation of quality-adjusted life-years by different preference-based instruments Med Care 2003 41 7 791 801 10.1097/00005650-200307000-00003 12835603

[B33] Kopec JA Willison KD A comparative review of four preference-weighted measures of health-related quality of life J Clin Epidemiol 2003 56 4 317 325 10.1016/S0895-4356(02)00609-1 12767408

[B34] Podsiadlo D Richardson S The timed "Up & Go": a test of basic functional mobility for frail elderly persons J Am Geriatr Soc 1991 39 2 142 148 1991946 10.1111/j.1532-5415.1991.tb01616.x

[B35] Oostenbrink JB Al MJ The analysis of incomplete cost data due to dropout Health Econ 2005 14 8 763 776 10.1002/hec.966 15729743

[B36] Ostbye T Crosse E Net economic costs of dementia in Canada Cmaj 1994 151 10 1457 1464 7954140 PMC1337410

[B37] Kesse-Guyot E Castetbon K Estaquio C Czernichow S Galan P Hercberg S Association between the French nutritional guideline-based score and 6-year anthropometric changes in a French middle-aged adult cohort Am J Epidemiol 2009 170 6 757 765 10.1093/aje/kwp174 19656810

[B38] Iersel MBv Kessels RPC Bloem BR Verbeek ALM Olde Rikkert MGM Executive Functions Are Associated With Gait and Balance in Community-Living Elderly People J Gerontol A Biol Sci Med Sci 2008 63 12 1344 1349 19126847 10.1093/gerona/63.12.1344

[B39] Liu-Ambrose T Katarynych LA Ashe MC Nagamatsu LS Hsu CL Dual-task gait performance among community-dwelling senior women: the role of balance confidence and executive functions J Gerontol A Biol Sci Med Sci 2009 64 9 975 982 19429702 10.1093/gerona/glp063

[B40] Stoehr GP Lu SY Lavery L Bilt JV Saxton JA Chang CC Ganguli M Factors associated with adherence to medication regimens in older primary care patients: the Steel Valley Seniors Survey Am J Geriatr Pharmacother 2008 6 5 255 263 10.1016/j.amjopharm.2008.11.001 19161928 PMC2675171

[B41] Kantor B Mauger L Richardson VE Unroe KT An analysis of an older driver evaluation program J Am Geriatr Soc 2004 52 8 1326 1330 10.1111/j.1532-5415.2004.52363.x 15271121

[B42] Johnson DR Emotional attention set-shifting and its relationship to anxiety and emotion regulation Emotion 2009 9 5 681 690 10.1037/a0017095 19803590

[B43] Weiner DK Rudy TE Morrow L Slaboda J Lieber S The relationship between pain, neuropsychological performance, and physical function in community-dwelling older adults with chronic low back pain Pain Med 2006 7 1 60 70 10.1111/j.1526-4637.2006.00091.x 16533199

[B44] West RL An application of prefrontal cortex function theory to cognitive aging Psychol Bull 1996 120 2 272 292 10.1037/0033-2909.120.2.272 8831298

[B45] Kuo H-K Lipsitz LA Cerebral White Matter Changes and Geriatric Syndromes: Is There a Link? J Gerontol A Biol Sci Med Sci 2004 59 8 M818 826 10.1093/gerona/59.8.m818 15345732

[B46] Colcombe S Kramer AF Fitness effects on the cognitive function of older adults: a meta-analytic study Psychol Sci 2003 14 2 125 130 10.1111/1467-9280.t01-1-01430 12661673

[B47] Lawton MP Brody EM Assessment of older people: self-maintaining and instrumental activities of daily living Gerontologist 1969 9 3 179 186 5349366

[B48] Chan SW Chiu HF Chien WT Goggins W Thompson D Hong B Predictors of change in health-related quality of life among older people with depression: a longitudinal study Int Psychogeriatr 2009 21 6 1171 1179 10.1017/S1041610209990950 19781111

[B49] Devlin N Parkin D Brown J Using the EQ-5D as a performance measurement tool in the NHS 2009

[B50] Cahn-Weiner DA Boyle PA Malloy PF Tests of executive function predict instrumental activities of daily living in community-dwelling older individuals Appl Neuropsychol 2002 9 3 187 191 10.1207/S15324826AN0903_8 12584085

[B51] Carlson MC Fried LP Xue QL Bandeen-Roche K Zeger SL Brandt J Association between executive attention and physical functional performance in community-dwelling older women J Gerontol B Psychol Sci Soc Sci 1999 54 5 S262 270 10542828 10.1093/geronb/54b.5.s262

[B52] Jefferson AL Paul RH Ozonoff A Cohen RA Evaluating elements of executive functioning as predictors of instrumental activities of daily living (IADLs) Arch Clin Neuropsychol 2006 21 4 311 320 10.1016/j.acn.2006.03.007 16814980 PMC2746400

[B53] Wlodarczyk JH Brodaty H Hawthorne G The relationship between quality of life, Mini-Mental State Examination, and the Instrumental Activities of Daily Living in patients with Alzheimer's disease Arch Gerontol Geriatr 2004 39 1 25 33 10.1016/j.archger.2003.12.004 15158578

[B54] Altman DG Bland JM Absence of evidence is not evidence of absence BMJ 1995 311 7003 485 7647644 10.1136/bmj.311.7003.485 PMC2550545

[B55] Marra CA Esdaile JM Guh D Kopec JA Brazier JE Koehler BE Chalmers A Anis AH A comparison of four indirect methods of assessing utility values in rheumatoid arthritis Med Care 2004 42 11 1125 1131 10.1097/00005650-200411000-00012 15586840

[B56] Peri K Kerse N Robinson E Parsons M Parsons J Latham N Does functionally based activity make a difference to health status and mobility? A randomised controlled trial in residential care facilities (The Promoting Independent Living Study; PILS) Age Ageing 2008 37 1 57 63 10.1093/ageing/afm135 17965045

[B57] Syddall HE Martin HJ Harwood RH Cooper C Aihie Sayer A The SF-36: a simple, effective measure of mobility-disability for epidemiological studies J Nutr Health Aging 2009 13 1 57 62 10.1007/s12603-009-0010-4 19151909 PMC2654814

[B58] Teixeira LE Silva KN Imoto AM Teixeira TJ Kayo AH Montenegro-Rodrigues R Peccin MS Trevisani VF Progressive load training for the quadriceps muscle associated with proprioception exercises for the prevention of falls in postmenopausal women with osteoporosis: a randomized controlled trial Osteoporos Int 2009 21 4 589 96 10.1007/s00198-009-1002-2 19562243

